# Human coronavirus 229E encodes a single ORF4 protein between the spike and the envelope genes

**DOI:** 10.1186/1743-422X-3-106

**Published:** 2006-12-28

**Authors:** Ronald Dijkman, Maarten F Jebbink, Berry Wilbrink, Krzysztof Pyrc, Hans L Zaaijer, Philip D Minor, Sally Franklin, Ben Berkhout, Volker Thiel, Lia van der Hoek

**Affiliations:** 1 Laboratory of Experimental Virology, Department of Medical Microbiology, Center for Infection and Immunity Amsterdam (CINIMA), Academic Medical Center, University of Amsterdam, The Netherlands; 2 Laboratory for Infectious Diseases and Screening, National Institute for Public Health and the Environment, Bilthoven, The Netherlands; 3 Laboratory of Clinical Virology, Department of Medical Microbiology, Center for Infection and Immunity Amsterdam (CINIMA), Academic Medical Center, University of Amsterdam, The Netherlands; 4 National Institute for Biological Standards and Controls (NIBSC), Hertfordshire, UK; 5 Kantonal Hospital St. Gallen, Research Department, St Gallen, Switzerland

## Abstract

**Background:**

The genome of coronaviruses contains structural and non-structural genes, including several so-called accessory genes. All group 1b coronaviruses encode a single accessory protein between the spike and envelope genes, except for human coronavirus (HCoV) 229E. The prototype virus has a split gene, encoding the putative ORF4a and ORF4b proteins. To determine whether primary HCoV-229E isolates exhibit this unusual genome organization, we analyzed the ORF4a/b region of five current clinical isolates from The Netherlands and three early isolates collected at the Common Cold Unit (CCU) in Salisbury, UK.

**Results:**

All Dutch isolates were identical in the ORF4a/b region at amino acid level. All CCU isolates are only 98% identical to the Dutch isolates at the nucleotide level, but more closely related to the prototype HCoV-229E (>98%). Remarkably, our analyses revealed that the laboratory adapted, prototype HCoV-229E has a 2-nucleotide deletion in the ORF4a/b region, whereas all clinical isolates carry a single ORF, 660 nt in size, encoding a single protein of 219 amino acids, which is a homologue of the ORF3 proteins encoded by HCoV-NL63 and PEDV.

**Conclusion:**

Thus, the genome organization of the group 1b coronaviruses HCoV-NL63, PEDV and HCoV-229E is identical. It is possible that extensive culturing of the HCoV-229E laboratory strain resulted in truncation of ORF4. This may indicate that the protein is not essential in cell culture, but the highly conserved amino acid sequence of the ORF4 protein among clinical isolates suggests that the protein plays an important role in vivo.

## Background

Coronaviruses (CoVs) are enveloped, plus-strand RNA viruses belonging to the family *Coronaviridae *[1]. The genomic RNA is 27 – 32 Kb in size, capped and polyadenylated. The virions are 80 – 150 nm in diameter and have a unique morphology, with extended, petal-shaped spikes that give the virus a crown-like projection (Latin; *corona*) under the electron microscope [1]. CoVs are classified into three groups based on phylogenetic and serological relationships. Group 1 and 2 consist of different mammalian coronaviruses, whereas bird viruses dominate group 3. All coronaviruses employ a common genome organization where the replicase gene encompasses the 5'-two thirds of the genome and is comprised of two overlapping open reading frames (ORFs), ORF1a and ORF1b. The structural gene region, which covers the 3'-third of the genome, encodes the canonical set of structural protein genes in the order 5'-spike (S) – envelope (E) – membrane (M) and nucleocapsid (N) – 3'. Expression of the replicase gene is mediated by translation of the genomic RNA that gives rise to the biosynthesis of two large polyproteins, pp1a (encoded by ORF1a) and pp1ab (encoded by ORF1a and ORF1b using a ribosomal frameshift at the ORF1a/1b junction). Expression of the structural gene region is mediated via discontinuous transcription of subgenomic (sg) mRNAs, a hallmark of coronavirus gene expression. The number of sg mRNAs produced by a particular coronavirus usually exceeds the number of encoded structural proteins and, consequently, coronaviruses are able to express additional, so-called – accessory – genes (formerly called group-specific genes). These genes are interspersed between the structural genes and their number and location varies within coronavirus genomes. The functions of coronavirus accessory proteins are largely unknown, however, reverse genetic analyses of Mouse Hepatits Virus (MHV) and Feline Infectious Peritonitis Virus (FIPV) suggest that they are not required for virus replication [2-4]. Moreover, deletion of MHV and FIPV accessory genes results in attenuation in their respective hosts, indicating that accessory genes represent pathogenicity factors [2-4].

**Figure 1:**
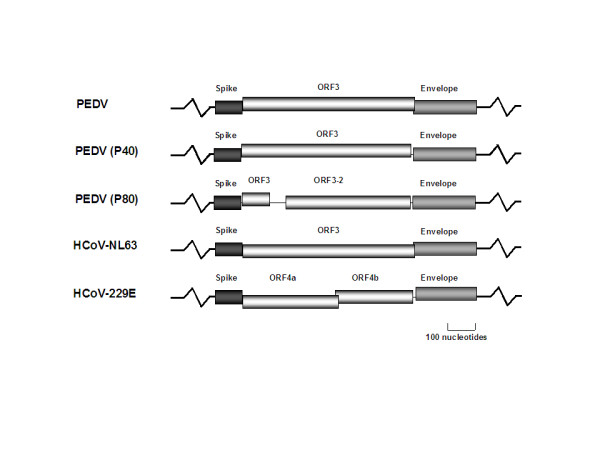
***Schematic overview of group 1b accessory protein genes between the S and E gene***. PEDV (NC_003436), HCoV-NL63 (NC_005831) and HCoV-229E (NC_002645), the truncated forms of PEDV (P40) and (P80) are based on previous published data [7].

The group 1 coronaviruses can be divided into the two genetic subgroups 1a and 1b [5]. Members of group 1a include canine coronavirus, FIPV, transmissible gastroenteritis virus (TGEV), and ferret enteric coronavirus. Group 1b includes porcine epidemic diarrhea virus (PEDV), human coronavirus NL63 (HCoV-NL63) and human coronavirus 229E (HCoV-229E). All members of group 1b encode one or two accessory proteins between the S and E gene, ORF3 protein for PEDV and HCoV-NL63, and ORF4a and ORF4b proteins for HCoV-229E (Figure 1). The numbering of the ORFs in HCoV-229E is based on Northern blot analysis of sg RNAs [6]. The presence of an additional sg mRNA in HCoV-229E-infected cells (i.e. sg mRNA3) shifts the numbering from ORF3 to ORF4a/b. However, the location of HCoV-229E ORFs 4a and 4b genes in the genome (i.e. between S and E) and sequence similarities to the group 1b ORF3 genes strongly support the notion that they are homologous. Unfortunately, very little information is currently available about the structure and function of the ORF3 proteins. Several studies have linked the ORF3 protein of PEDV and TGEV to viral infectivity and pathogenicity [7,8]. PEDV and TGEV acquire truncated forms of their accessory proteins after extensively passaging in cell culture, and these laboratory-adapted strains, encoding truncated forms of ORF3-proteins, are less pathogenic than the corresponding wild-type strains (Figure 1) [7,8].

HCoV-229E contains two ORFs, ORF4a and ORF4b between the S and E genes (Figure 1). Since both genes share the same sg mRNA (i.e. sg mRNA4), the expression of gene 4b would require alternative mechanisms of translation, such as internal entry, leaky scanning, or translational reinitiation of ribosomes. However, comparison of the hydrophobic domains of both ORF4 parts with the single ORF3 homologs indicates that they encode a similar protein [9], suggesting a scenario in which HCoV-229E acquired an out-of-frame insertion or deletion. It should be noted that the origin of full-length genomic sequences of the group 1b coronaviruses PEDV and HCoV-NL63 are derived from clinical isolates, CV777 and Amsterdam-1, Amsterdam-057 and Amsterdam-496, respectively. In contrast, the HCoV-229E ORF4a/b sequence [10] and the HCoV-229E full-length genomic sequence [6] has been determined from a cell culture-adapted virus more than 30 years after the initial isolation of HCoV-229E by Hamre and Procknow [11]. We, therefore, hypothesized that HCoV-229E ORFs 4a/b might actually had been a single ORF that was truncated upon adaptation of HCoV-229E to cell culture.

## Results

### Analysis of HCoV-229E cell culture adapted virus

**Figure 2:**
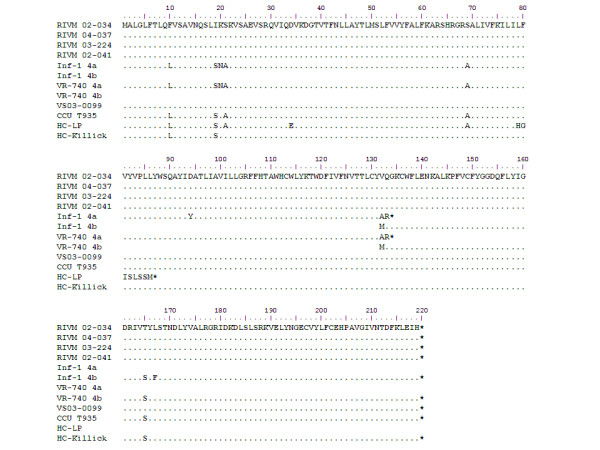
***Amino acid sequence alignment of ORF4 of laboratory-adapted and clinical HCoV-229E strains***. "." Denotes identical amino acid to the RIVM 04-037 sequence; "*" denotes stopcodon.

We first analyzed the ORF 4a/b region of VR-740™, a cell culture-adapted virus that also originates from the initial HCoV-229E clinical isolate and which was deposited at the American Type Culture Collection (ATCC) in 1973. The ORF4a/b sequence of VR-740™ showed an overall 99% similarity at the nucleotide level with the published sequence of the cell culture-adapted HCoV-229E, and we detected only one single amino acid substitution in each protein (ORF4a protein; aa 94 Y>D, ORF4b protein; aa 36 F>L). Still, like the cell culture-adapted HCoV-229E, VR-740™ encodes two ORFs between the S and E genes (Figure 2).

### Analysis of current clinical HCoV-229E isolates

The apparent identity of the ORF4a/b region in both cell culture-adapted viruses prompted us to elucidate this region in clinical HCoV-229E isolates, of which no ORF4a/b sequence data is available thus far. We therefore studied nose/throat swab and nasopharyngeal aspirate materials from 5 patients that were tested positive for HCoV-229E. The clinical symptoms of the HCoV-229E-infected patients were similar to those that are commonly observed for HCoV-229E infections, with symptoms like rhinorrhoea, fever and malaise (Table 1) [12]. RT-PCR sequencing analysis of the ORF4a/b region revealed an overall nucleotide similarity of 97% of the Dutch clinical isolates to that of the cell culture-adapted viruses. Of most interest is the presence of a 2-nucleotide deletion, within the ORF4a gene of the cell culture-adapted viruses when aligned to the Dutch isolates. Strikingly, this Thymidine and Guanosine deletion at position 395–396 is absent in all clinical isolates. Deduced protein sequences encoded by ORF4a and ORF4b from the published HCoV-229E sequence, the sequence of VR-740™, determined in this study, and the sequences of the 5 clinical isolates were aligned. This reveals that clinical isolates encode a single, uninterrupted ORF between the S and E genes (Figure 2). This single accessory gene is 660 nucleotides in length and encodes an ORF4 protein that is 219 amino acids in size, with a high similarity of the N- and C-terminal domains with ORF4a (93%) and ORF4b (96%), respectively. All Dutch sequences have the same amino acid sequence (Figure 2), but some silent mutations were observed at the nucleotide level, excluding the possibility of PCR contamination.

**Table 1:** Clinical symptoms of HCoV-229E infected patients.

			**Symptoms**								
**Patient**	**Age**	**Year of Sampling**	**Acute start**	**Cough**	**Rhinorrhoea**	**Sore Throat**	**Fever**	**Malaise**	**Other**	**Hospitalized**	**Diagnosis**

RIVM 02-034	52	2002	Yes	No	Yes	No	No	Yes	Muscle/joint pain	No	ARI, Pharyngitis
RIVM 02-041	54	2002	Yes	No	Yes	No	Yes	No	Hoarse	No	Common Cold, Acute tonsillitis
RIVM 03-224	68	2003	Yes	No	Yes	Yes	Yes	No	Respiratory allergy	No	Common Cold
RIVM 04-037	54	2004	Yes	Yes	Yes	Yes	Yes	Yes	-	No	ARI
VS03-099*	81	2003	Yes	Yes	U	U	No	Yes	Dyspnoea	Yes	Exacerbation of COPD
CCU-T935^†^	U	1986	U	U	U	U	U	U	U	U	U

*The person was on treatment for an atypical Mycobacterium infection.

^†^Unfortunately, all administration on Trail No T935 is no longer available.

U = Unknown

ARI = Acute Respiratory infection

COPD = Chronic Obstructive Pulmonary Disease

### Analysis of early HCoV-229E isolates

**Figure 3:**
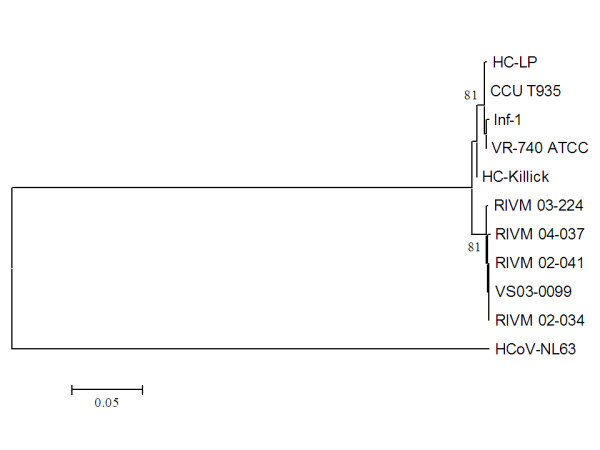
***Phylogenetic analyses of ORF4 nucleotide sequences of laboratory-adapted and clinical HCoV-229E strains***. ORF3 of HCoV-NL63 (NC_005831) was used as outgroup; bootstrap values above 70 are shown.

Although our data suggest that the deletion in the laboratory HCoV-229E strain and VR-740™ is the result of cell culture-adaptation, we cannot rule out the possibility that the deletion might represent natural variation between different HCoV-229E isolates. Since the VR-740™ virus was deposited at the ATCC in 1973, HCoV-229E might have evolved over the years and current clinical isolates may thus differ from the prototype. Alternatively, the Dutch clinical isolates may represent a subgroup of HCoV-229E, and VR-740™-like clinical isolates may exist as a second HCoV-229E subgroup. To investigate whether the 2-nucleotide deletion is also present in isolates that are more related to VR-740™, we searched for other early HCoV-229E isolates. We were able to retrieve three early isolates that were originally collected at the common cold unit (CCU), Salisbury, UK. Two of these are laboratory-adapted viruses of which it is unknown in which extend they were passaged. HC-LP was initially isolated in 1965 [13] and HC-Killick in the eighties [14]. The third sample, CCU-T935, was obtained from an infected person that participated trial T935 in 1986, of which unfortunately all data were lost. Phylogenetic analysis shows that the sequences of ORF4a/b from the three early isolates were indeed more related to the cell culture-adapted, prototype viruses (98 – 99%) than to the current Dutch isolates (98%) (Figure 2 and 3). Most importantly, however, the early isolate sequences of CCU-T935 and HC-Killick, like those derived from the clinical isolates, do not contain the 2-nucleotide deletion, but carry the uninterrupted ORF between S and E. Interestingly, HC-LP does not have this particlar 2-nucleotide deletion, instead, a larger (118 nt) deletion was observed that is located halfway the ORF4a region (Figure 2).

## Discussion

Hypothetically, an ORF4b protein could be translated via alternative translation mechanisms, as described for some other coronavirus proteins [15,16], but those mechanisms have not been described for this region, nor has any evidence for the expression of HCoV-229E ORF4b protein ever been reported. In addition, our results show that a large fragment is deleted in the cell-culture adapted HC-LP virus, which corresponds with the ORF4b region of the prototype virus. As mentioned previously, it has been shown for MHV and FIPV that accessory genes are dispensable for virus growth in cell culture. Moreover, the deletion of accessory genes resulted in these cases in viruses that are attenuated in vivo. Similarly, attenuation of in vivo viral infectivity and pathogenicity has been linked to ORF3 truncation upon in vitro culturing of other group 1b coronaviruses. For a virulent PEDV strain this occurred after 40 passages, and more severe truncation and attenuation was observed after 60 or more passages [7]. Similar results have been reported for TGEV after at least 35 passages [8]. Unfortunately, no detailed information is available about the in vitro passaging of the cell culture-adapted HCoV-229E strains. It is tempting to speculate that ORF4 of HCoV-229E, like ORF3 of PEDV, is vital for efficient in vivo replication. The fact that VR-740™ contains a truncated ORF4 may explain why this virus replicates in vitro in murine cells expressing HCoV-229E receptor (human CD13), but not in vivo in the human CD13 transgenic animals [17,18]. It is of interest to investigate whether an HCoV-229E strain with a more severe truncated or a non-truncated ORF4 gene can replicate in these mice.

Accompanied with the deletions, we also observed several non-silent nucleotide differences between the cell culture-adapted viruses and the clinical isolates. In our Dutch isolates the ORF4 is highly conserved on the protein level. The CCU T935 isolate that was collected in 1986 at the CCU, Salisbury, is a clinical isolate with high ORF4 similarity to the cell culture-adapted viruses. Since we cannot reconstruct the experimental setting performed during the clinical trail T935 at the CCU in 1986, we cannot exclude the possibility that the CCU T935 sample was obtained from a volunteer inoculated with an HCoV-229E laboratory strain. This strain might even have the same origin as the cell culture-adapted viruses [11]. In any case, the CCU T935 sample is derived from an "in vivo" infection, be it experimental or natural, and this further supports a relevant in vivo function of HCoV-229E full-length ORF4 protein. We believe that the divergence between the current Dutch isolates and the early CCU T935 strain most likely represents genetic drift over 20 – 30 years of evolution [19]. Molecular clock analysis with the average mutation rate of coronaviruses [20,21] supports this idea (data not shown). Given the long time of evolution the differences between the CCU T935 and Dutch isolates are remarkably small. For HCoV-NL63 we also observed a highly conserved ORF3 among different clinical isolates [22], and although for PEDV limited sequence data are available, Song *et al*. found only one nucleotide difference in ORF3 between two PEDV field isolates [7].

Recently, Tang *et al*. reported on novel bat coronaviruses (Bt-CoVs), of which several cluster with group 1b coronaviruses. They determined the full-length genomic sequence of one of these wild-type Bt-CoVs (Bt-CoV/512/2005) [23]. The genome organization of this Bt-CoV strain is similar to that of the other group 1b members, with the exception of one putative gene at the 3'end of the genome. However, only one accessory protein, encoded by ORF3, is identified between the structural genes S and E. The ORF3 protein of Bt-CoV/512/2005 is homologous to ORF3 proteins of PEDV, HCoV-NL63 and the ORF4 protein from our clinical HCoV-229E isolates. These data show that all currently sequenced group 1b coronaviruses contain one homologous accessory gene between the S and E genes.

## Conclusion

We report the first sequences of the ORF4a/b region of clinical HCoV-229E isolates. The experimental data strongly support the hypothesis that a separation of a formerly single ORF4 had taken place upon adaptation of HCoV-229E to cell culture. We observed two different types of deletions, 2 or 118 nucleotides, of the ORF4 gene only in cell culture-adapted viruses whereas all clinical isolates, including CCU T935, encoded a single ORF4 gene. Both types of nucleotide deletion within the ORF4a/b region of cell culture-adapted HCoV-229E viruses creates a frame shift that introduces an early termination codon, which either separates ORF4 to ORF4a and ORF4b or results in a truncated ORF4(a) fragment (HC-LP). Most likely, the two types of deletion occurred independently and are not site specific. Therefore the genome organization for the group 1b coronaviruses (HCoV-NL63, PEDV, Bt-CoV *and *HCoV-229E) is identical. The amino acid sequence of the HCoV-229E ORF4 protein is highly conserved among clinical isolates suggesting that the protein plays an important role during in vivo infection.

## Methods

### Collection of patient material

Patient materials were collected at the department of Medical Microbiology, Academic Medical Center (AMC), The Netherlands (VS03-099) and from the Laboratory for Infectious Diseases and Screening, National Institute of Public Health and the Environment (RIVM), Bilthoven, The Netherlands (RIVM02-034, RIVM02-041, RIVM03-224 and RIVM04-037) (Table 1). One sample was collected in 1986 at the common cold unit (CCU), Salisbury, Great Britain, during Trail no. T935.

### Viral RNA isolation

Total viral RNA was isolated either from 200 μl cell culture supernatant, 100 – 200 μl nose/throat swab (RIVM) or nasopharyngeal aspirate (AMC) as previously described [24].

### RT-PCR

Reverse transcription and PCR reactions were performed as described [22,25]. Amplification of the ORF4a/b region was performed with the primer combination 5'-229E-ORF4ab (5'-AAC TTC CTT ATT ACG ACG TT-'3) and 3'-229E-ORF4ab (5'-ATC CAC TAG CTT AAG GAA CA-'3). If required, a semi-nested PCR was performed with the primers 5'-229E-ORF4abNested (5'-CAT ACA GTA ATG GCT CTA GG-'3) and 3'-229E-ORF4ab, and the cycle profile of the first PCR was modified to 30 cycles.

### Sequence analysis of ORF4a/b region

RT-PCR fragments were directly sequenced with primers 3'-229E-ORF4ab, 5'-229E-ORF4ab, 5'-229E-ORF4abNested, 5'229E4int (5'-GCA ACT TTG ATT GCT G-'3) and 3'229E4int (5'-GTC CTC TAA GAG CAA C-'3). Sequence reaction was preformed without purifying steps, according to the BigDye^® ^terminator V1.1 cycle sequencing manufacturer's protocol on a GeneAmp^® ^PCR System 9700 thermal cycler (Perkin Elmer). Electrophoresis and data collection was performed on a 3100 Genetic Analyzer (Applied Biosystems). Raw collection data was processed and analyzed with Codoncode Aligner v1.52 software (CodonCode Corporation).

### Deduced protein sequences

Deduced protein sequences encoded by ORF4a and ORF4b from the published HCoV-229E sequence (ORF4a; NP_073552, ORF4b; NP_073553), the sequence of VR-740™, determined in this study, and the sequences of the 5 current and the 3 early isolates were aligned with ClustalX v1.8, and manually adjusted with Bioedit v7.0.1.

### Phylogenetic analysis of the ORF4a/b region

The sequences of the ORF4a/b regions were aligned with ClustalX v1.8 and phylogenetic analyses was conducted with the neighbor-joining method, Kimura distances and a bootstrap of 1000 replicates, using MEGA version 3.1 [26].

### Data deposition

The sequences reported in this paper have been deposited under the Genbank database accession numbers EF198671–EF198679.

## Competing interests

The author(s) declare that they have no competing interests.

## Authors' contributions

RD carried out the viral RNA isolation, RT-PCR, sequencing of RT-PCR fragments and computer analysis. MFJ carried out the design and testing of primers and RT-PCR. BW, HLZ, PDM and SF carried out the collection of the patient material and the clinical data. KP carried out the molecular clock analysis; all authors participated in writing the manuscript. LvdH, BB and VT are the principal investigators.
